# Two functional variants at 6p21.1 were associated with lean mass

**DOI:** 10.1186/s13395-019-0212-3

**Published:** 2019-11-23

**Authors:** Yu-Fang Pei, Wen-Zhu Hu, Xiao-Lin Yang, Xin-Tong Wei, Gui-Juan Feng, Hong Zhang, Hui Shen, Qing Tian, Hong-Wen Deng, Lei Zhang

**Affiliations:** 1 Department of Epidemiology and Health Statistics, School of Public Health, Medical College, SuZhou City, People’s Republic of China; 2 Jiangsu Key Laboratory of Preventive and Translational Medicine for Geriatric Diseases, Soochow University, SuZhou City, People’s Republic of China; 3 School of Public Health, Southeast University, Nanjing, People’s Republic of China; 4 Center for Genetic Epidemiology and Genomics, School of Public Health, Medical College, Soochow University, 199 Ren-ai Rd., SuZhou City, 215123 Jiangsu Province, People’s Republic of China; 5 Department of Biostatistics and Bioinformatics, Tulane University School of Public Health and Tropical Medicine, New Orleans, LA USA

**Keywords:** Genome-wide association study, Lean body mass, 6p21.1, *NFKBIE*

## Abstract

**Background:**

Low lean body mass is the most important predictor of sarcopenia with strong genetic background. The aim of this study was to uncover genetic factors underlying lean mass development.

**Materials and methods:**

We performed a genome-wide association study (GWAS) of fat-adjusted leg lean mass in the Framingham Heart Study (FHS, *N* = 6587), and replicated in the Women’s Health Initiative–African American sub-sample (WHI-AA, *N* = 847) and the Kansas City Osteoporosis Study (KCOS, *N* = 2219). We also cross-validated significant variants in the publicly available body mass index (BMI) summary results (*N* ~ 700,000). We then performed a series of functional investigations on the identified variants.

**Results:**

Four correlated SNPs at 6p21.1 were identified at the genome-wide significance (GWS, *α* = 5.0 × 10^−8^) level in the discovery FHS sample (rs551145, rs524533, rs571770, and rs545970, *p* = 3.40–9.77 × 10^−9^), and were successfully replicated in both the WHI-AA and the KCOS samples (one-sided *p* = 1.61 × 10^−3^–0.04). They were further cross-validated by the large-scale BMI summary results (*p* = 7.0–9.8 × 10^−3^). Cis-eQTL analyses associated these SNPs with the *NFKBIE* gene expression. Electrophoresis mobility shift assay (EMSA) in mouse C2C12 myoblast cells implied that rs524533 and rs571770 were bound to an unknown transcription factor in an allelic specific manner, while rs551145 and rs545970 did not. Dual-luciferase reporter assay revealed that both rs524533 and rs571770 downregulated luciferase expression by repressing promoter activity. Moreover, the regulation pattern was allelic specific, strengthening the evidence towards their differential regulatory effects.

**Conclusions:**

Through a large-scale GWAS followed by a series of functional investigations, we identified 2 correlated functional variants at 6p21.1 associated with leg lean mass. Our findings not only enhanced our understanding of molecular basis of lean mass development but also provided useful candidate genes for further functional studies.

## Background

Sarcopenia is a complex aging disorder [1]. According to Baumgartner et al. [2], 13–24% of the elderly white people under 70 years old have sarcopenia, and the prevalence is as high as 50% or more among those aged 80 years or older. The diagnosis of sarcopenia is based on an assessment of skeletal muscle mass and/or muscle strength, as well as muscle functions [3]. Among them, lean mass, as measured by dual-energy X-ray absorptiometry (DXA) scan, is regarded as the most important predictor of sarcopenia [4, 5]. The decline in the contractility of lean mass also leads to a decrease in the load on bones, which makes the bones in a state of disuse for a long time, and is easy to induce osteoporosis [6]. A series of negative consequences caused by sarcopenia on behavioral and physiological functions, including falls, fracture, and osteoarthropathy, could lead to high rates of hospitalizations and mortality especially in the elderly [7].

Despite being influenced by environmental factors including life style and endocrine changes, sarcopenia is mainly dominated by inheritance. The heritability of muscle strength varies from 30 to 85%, while that of lean mass achieves as high as 45–90% [8, 9]. Several genome-wide association studies (GWAS) have been performed for sarcopenia related traits. In 2009, Liu et al. conducted a single sample GWAS in 1000 white people and identified one gene thyrotropin-releasing hormone receptor (*TRHR*) [10]. Since then, almost 20 susceptible loci have been identified by a series of GWAS and their meta-analyses [10–13]. Nonetheless, the identified loci explain only a small portion of phenotypic variation, and the majority of hidden heritability is yet to be identified.

In the present study, aiming to achieve a better understanding of the genetic etiology of sarcopenia, we conducted a genome-wide association study of leg lean mass using the Framingham Heart Study (FHS) as discovery sample and two independent samples as replication samples. In addition, we conducted a series of bioinformatic analysis and biological experiments, including cis-eQTL analysis, electrophoretic mobility shift assay (EMSA), and dual-luciferase reporter assay, to explore the functional relevance of the identified variants.

## Materials and methods

Two samples used in this study, including the FHS and the Women’s Health Initiative study–African American (WHI-AA) sub-sample, were accessed through the database of genotype and phenotype (dbGAP) portal. Neither phenotyping nor genotyping in these samples was performed by the authors. Instead, both phenotyping and genotyping in the third sample, the Kansas City Osteoporosis Study (KCOS), were done by the authors.

Ethics approval was obtained from local institutional review boards of all institutions, and all participants gave written informed consent before being recruited into the study.

### Discovery sample

Details of the study design and sample recruitment of the discovery FHS sample have been described elsewhere [14]. Briefly, the FHS is a longitudinal and prospective cohort comprising > 16,000 pedigree participants spanning three generations of European ancestry. The original generation consisted of 5209 participants living in the town of Framingham, MA, USA. The offspring generation consisted of 5124 participants who were adult children of the original cohort members or were spouses of these offspring. The third generation consisted of over 4000 participants who were grandchildren of the original generation.

The original generation underwent bone densitometry scan by dual-energy X-ray absorptiometry (DXA, Lunar Corp., Madison, WI, USA) during their 22nd or 24th examination. The offspring participants underwent DXA scan during their 6th or 7th examination, and the third generation participants underwent DXA scan during their 2nd examination. Fat body mass (FBM) and total soft tissue body mass were measured by the DXA scanner. Lean body mass (LBM) was approximated by subtracting FBM from total soft tissue mass.

A subset of participants were genotyped by the Affymetrix high-throughput 500 K genotyping array plus a supplemental 50 K genotyping array. To maximize genotype coverage, we merged these two genotype data sets together to form a single dataset of ~ 550,000 genotyped SNPs.

After checking the availability of both genotyped and phenotyped participants, we identified a total of 6587 family members, of which 693, 2749 and 3145 were from the original, offspring, and third generations, respectively.

### Replication samples

The first replication sample is the WHI-AA sample, which is a sub-sample of the WHI observational study that was accessed through the dbGAP. The WHI study is a partial factorial randomized and longitudinal cohort with > 90,000 postmenopausal women aged 50–79 years from two US minority populations: African-American and Hispanic. Participants were recruited at 40 clinical centers across the USA [15]. LBM was measured by DXA bone densitometers (Hologic Inc., Bedford, MA, USA). Participants were genotyped by the Affymetrix SNP 6.0 genotyping array. The WHI-AA sample included 845 African-American participants who were available of both genotypes and phenotypes for analysis.

The second replication sample is the KCOS sample. The KCOS is a cross-sectional study of participants living in Kansas City, MO, USA, and its surrounding areas. A selected set of 2286 participants of European ancestry were genotyped by the Affymetrix SNP 6.0 genotyping array. The genotyped participants were normal healthy subjects defined by a comprehensive suite of exclusion criteria, as described elsewhere [16]. LBM was measured by a DXA bone densitometer (QDR 4500 W, Hologic Inc., Bedford, MA, USA), following the manufacturer’s protocol.

### Phenotype modeling

In the discovery FHS sample, covariates were screened among leg fat mass, gender, age, age squared, height and height squared with the stepwise linear regression model. Leg lean mass was adjusted by significant covariates. Moreover, the top 10 principal components derived from the genome-wide genotype data were included as covariates to account for potential population stratification. The residual was normalized by inverse quantiles of standard normal distribution. In the replication samples, raw lean mass was modeled in the same way.

### Genotype quality control

Quality control (QC) of genotype data within each sample was implemented at both individual and SNP levels. At the individual level, sex compatibility was checked by imputing sex from X-chromosome genotype data with PLINK [17]. Individuals of ambiguous imputed sex or of imputed sex inconsistent with reported sex were removed. At the SNP level, SNPs violating the Hardy–Weinberg equilibrium (HWE) rule (*p* value < 1.0 × 10^−5^) were removed. In the discovery FHS sample, genotypes presenting the Mendel error were set to missing. Population outliers were monitored by genotype-derived principal components, and were removed if present.

### Genotype imputation

The FHS sample was imputed by the 1000 Genomes Project sequencing data (as of May 2013) [18]. Firstly, phased variants of 503 individuals of European ancestry were downloaded from the 1000 Genomes Project website. Secondly, bi-allelic variants, including SNPs and bi-allelic deletion/insertion variants (DIVs), were extracted, forming a reference panel for imputation.

As a QC step, variants with zero or one copy of a minor allele were removed. Prior to imputation, a consistency test of allele frequency between the FHS sample and the reference sample was examined with the chi-square test. To correct for potential mis-strandedness, SNPs that failed the consistency test (*p* < 1.0 × 10^−6^) were transformed into the reverse strand in the FHS sample. SNPs that again failed the consistency test were removed from the FHS sample. Imputation was performed with FISH, a fast and accurate diploid genotype imputation algorithm developed by us [19]. One of the most prominent features of this algorithm is the ability to impute diploid genotypes instead of haploid haplotypes, while keeping the computation time linear to the size of reference panel.

The replication samples were imputed with the same procedure. For the WHI-AA sample, haplotypes representing 319 subjects of the African ancestry were used as reference panel. The imputation certainty was measured by the imputation score *r*^2^, which was defined as the correlation between imputed dosage and the best imputed genotype. Variants of low imputation score (*r*^2^ < 0.3) or of low frequency (MAF < 0.05) were excluded from subsequent analyses.

### Association testing

In the discovery FHS sample, genetic association between normalized phenotype residuals with genotyped and imputed genotypes was tested under an additive mode of inheritance. A mixed linear model was used to account for familial relatedness, in which the genotype effect was modeled as fixed effects while the family member relatedness was modeled as random effects [20]. Association test was examined within the variance-components framework.

Associations identified at the discovery sample were further examined in the replication samples by a linear regression model with MACH2QTL [21]. For each replication sample, we first checked if the effect direction was consistent between the discovery sample and the replication sample. Upon the consistent effect direction, we then reported one-sided replication *p* value. Significance threshold was set at the nominal level *α* = 0.05.

### Cis-eQTL analysis

To investigate the association between the identified SNP polymorphisms and the nearby gene expressions, we performed cis-eQTL analysis. We used the following two large-scale datasets for analysis. The first one is the GTEx project dataset [22]. The GTEx project collected and RNA-sequenced multiple human tissues (up to 11,614) from donors who were also densely genotyped, and analyzed associations between SNPs and global RNA expression within individual tissues. We downloaded the summary results of skeletal muscle tissue from the GTEx website (V7). The second one is the Westra et al.’s study [23]. This study performed an eQTL meta-analysis in 5311 peripheral blood subjects from 7 studies, and performed replication analysis in another 2775 subjects. We downloaded significant cis-eQTL results from the study website (http://www.genenetwork.nl/bloodeqtlbrowser/).

### Cell culture

The mouse C2C12 myoblast cells were purchased commercially (Procell Life Science&Technology, China). They were grown in Dulbecco’s modified Eagle’s medium (HyClone, USA) supplemented with 10% fetal bovine serum (Zhejiang Tianhang Biotechnology, China) and 100 U/mL penicillin as well as 100 μg/mL streptomycin (Beyotime Institute of Biotechnology, China). The culture condition was at 37 °C in a humidified incubator of 5% CO_2_.

### Electrophoretic mobility shift assay

EMSA was performed to explore transcription factor binding ability of the identified SNPs. For each SNP, a 93 bp oligonucleotide competitor probe containing either major or minor allele of the SNP was synthesized (Shanghai Sangon Biotech, China) (Additional file 1: Table S1). Labeled probe was generated by labeling the 3′- end of the competitor probe with biotin using the EMSA probe biotin labeling kit (Beyotime, China). The sequence was then synthesized into double-strand DNA (dsDNA) probe by PCR. The dsDNA probe and nuclear protein extracted from C2C12 cells were co-incubated at room temperature for 20 min in 1 × binding buffer using the Chemiluminescent EMSA kit (Beyotime, China). Incubation products were separated in 6% non-denaturing polyacrylamide gels in 0.5× Tris-borate-EDTA (TBE) at 100 volts (V). Subsequently, the probes were transferred to positively charged nylon membranes in 0.5× TBE at 60 V for 1 h. The membranes were treated by UV cross-linking and incubated with blocking liquid containing Streptavidin-HRP Conjugate (Beyotime, China) for 15 min. DNA-protein bands were visualized by the BeyoECL Plus (Beyotime, China) according to the manufacturer’s instructions.

For each sequence containing either major or minor allele, the following five conditions were conducted in total volume of 10 μL: (1) negative control, 1 μL of biotin-labeled probe (0.07 μM) was incubated with 2 μL of 5× binding buffer containing poly (dI-dC), a non-specific competitor of DNA. The mixture was incubated at 25 °C for 20 min; (2) binding reaction, before adding biotin-labeled probes at room temperature for 20 min, 5× binding buffer was pre-incubated with nuclear protein at 25 °C for 10 min; 3–5) competition reaction, prior to the addition of the labeled probe, three levels (2-, 10-, and 50-fold) of excess of unlabeled probe were added to the reaction mixture (5 × binding buffer and nuclear protein) for 20 min, respectively.

### Dual-luciferase reporter assay

We performed dual-luciferase reporter assay to examine the allelic specific effect of the identified SNPs on gene expression. For each SNP, a 213 bp human DNA sequence centering at the target SNP was synthesized (Shanghai Sangon Biotech, China) (Additional file 2: Table S2). Two types of sequence, each containing either major or minor allele of the target SNP, were synthesized. The synthesized sequence was inserted into the upstream of the promoter element of the pGL3-promoter reporter plasmid (Promega, USA), forming two constructs: pGL3-promoter-major allele and pGL3-promoter-minor allele. The success of plasmid construction was confirmed by DNA Sanger-sequencing. The construct was then transfected into the C2C12 cells by using the jetPRIME transfection reagent (PolyPlus-transfection, France). As an internal control for transfection efficiency, the pRL-TK vector (Promega Corporation, Madison, WI, USA) expressing renilla luciferase gene was co-transfected at the same time. Each sample was duplicated in four independent parallel wells. Luciferase activity was measured by dual-luciferase kit (Promega, USA) 24 h later and normalized according to the renilla luciferase activity. The relative luciferase activity values were analyzed using the unpaired 2-tail Student’s *t* test.

## Results

### Discovery sample

**Fig. 1:**
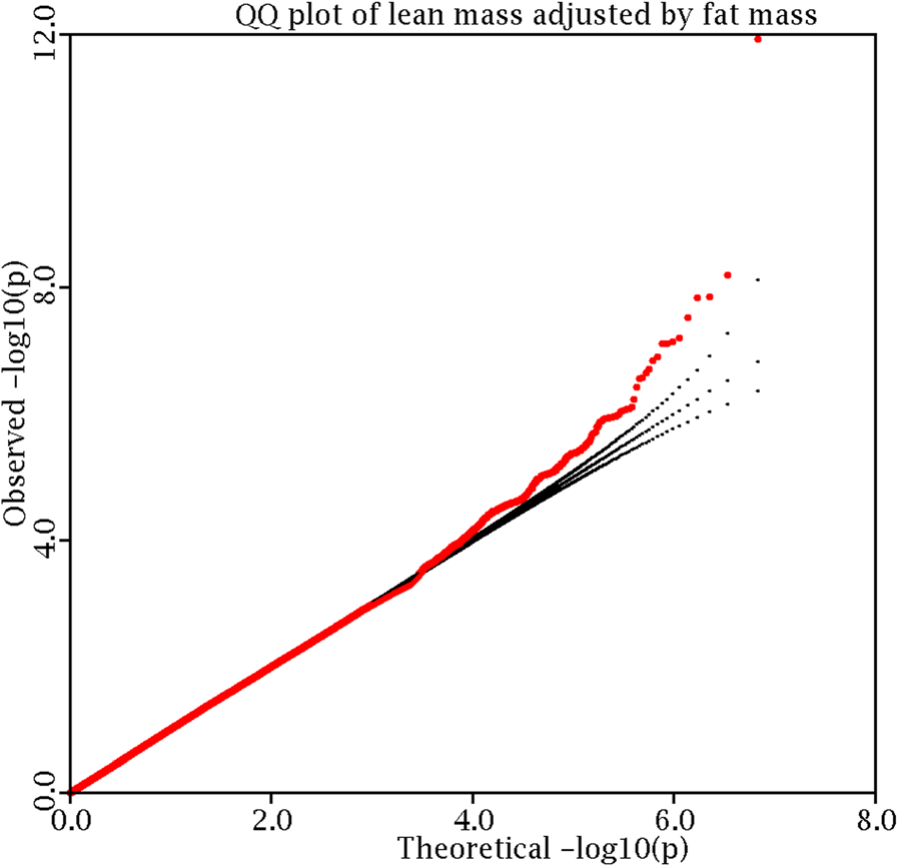
Logarithmic quantile–quantile (QQ) plot of the discovery GWAS *p* values. Ten-based logarithmic *p* value was plotted versus theoretical expectation (in red), while the theoretical expectation and its 95% confidence interval (CI) were plotted in dashed black line. The deviation from the theoretical expectation in the tail distribution implied the existence of positive association signals

Basic characteristics of the discovery sample are listed in Additional file 3: Table S3. A total of 6587 subjects are available for analysis; 55% of them are women. The 1000 Genomes Project generated 12,403,269 bi-allelic variants. After removing variants either of low-frequency or of poor imputation accuracy, 6,879,267 variants are qualified for analysis. Eighty-eight percent (6,035,487) of them are SNPs, and the remaining 12% (843,780) are DIVs. Genomic control inflation factor is 1.14. To correct for potential population stratification, we adjusted individual *p* values by the GC factor. A logarithmic quartile-quartile plot of the adjusted test statistics shows a marked deviation in the tail of the distribution, implying the possible existence of true associations (Fig. 1).

A total of 15 SNPs are associated with leg lean mass at the genome-wide significance (GWS, 5.0 × 10^−8^) level. Twelve of them are located at 6p21.1, one at 5q22.3, one at 9q21.13, and the last at 10q24.33. At 6p21.1, the lead SNP is rs513688 (beta = − 0.11, *p* = 5.61 × 10^−10^). There are only one SNP rs2004680 at 5q22.3, one SNP rs12351661 at 9q21.12, and one SNP rs17813699 at 10q24.33 significant at the GWS level (rs2004680 beta = − 0.09, *p* = 1.92 × 10^−8^; rs12351661 beta = − 0.13, *p* = 3.32 × 10−^14^; rs17813699 beta = − 0.09, *p* = 4.04 × 10^−8^), respectively. Manhattan plot of the discovery association results is displayed in Additional file 5: Figure S1.

### Replication samples

Basic characteristics of the replication samples are listed in Additional file 4: Table S4. The first replication sample is the WHI-AA sample (*N* = 847). Of the 12 significant SNPs at 6p21.1, 4 SNPs rs551145, rs524533, rs571770, and rs545970 are successfully replicated (rs551145 beta = 0.27, *p* = 2.25 × 10^−3^; rs524533 beta = − 0.23, *p* = 1.61 × 10^−3^; rs571770 beta = − 0.19, *p* = 5.97 × 10^−3^; rs545970 beta = − 0.11, *p* = 0.04, all one-sided) with the same effect direction to those in the discovery sample, while the 8 other SNPs including the lead one rs513688 are not nominally significant.

The only significant SNP rs2004680 at 5q22.3 is not replicated (*p* = 0.19), though the effect direction is consistent to that in the discovery sample. For the only significant SNP rs12351661 at 9q21.12, neither the *p* value significant (*p* = 0.42) nor the effect direction is consistent. Finally, the only significant SNP rs17813699 at 10q24.33 is not available in the WHI-AA sample.

The second replication sample is the KCOS sample of the European ancestry (*N* = 2219). At 6p21.1, all the above 4 replicated SNPs are successfully replicated by the KCOS sample (rs551145 beta = 0.08, *p* = 8.49 × 10^−3^; rs524533 beta = − 0.07, *p* = 0.02; rs571770 beta = − 0.07, *p* = 0.01; rs545970 beta = − 0.07, *p* = 0.02, all one-sided). In addition, the other 5 significant SNPs including the lead one rs513688 (one sided *p* = 0.01) are also nominally significant with the same effect direction. None of the SNPs at the other 3 loci is significant.

Combining the evidence from both the discovery and replication samples, the locus 6p21.1 is convincingly associated with leg lean mass after adjustment by leg fat mass. The 4 SNPs rs551145, rs524533, rs571770, and rs545970 at this locus are significant at the GWS level in the discovery FHS sample, and are nominally significant in both the WHI-AA sample and the KCOS sample. Main association results are listed in Table 1.

**Table 1:** Main association results of leg lean mass at 6p21.1

SNP	Position	Alleles	EAF	FHS (*N* = 6587)		WHI-AA (*N* = 847)		KCOS (*N* = 2219)		GIANT+UKB (BMI)		
				Beta	*p*	Beta	p*	Beta	p*	Beta	*p*	*N*
rs551145	44237448	C/T	0.75	0.10	*3.40 × 10*^*−9*^	0.27	2.25 × 10^−3^	0.08	8.49 × 10^−3^	-	-	-
rs545970	44239677	C/G	0.31	− 0.10	*7.46 × 10*^*−9*^	− 0.11	0.04	− 0.07	0.02	− 0.01	7.00 × 10^−3^	634887
rs571770	44240193	C/G	0.31	− 0.10	*9.02 × 10*^*−9*^	− 0.19	5.97 × 10^−3^	− 0.07	0.01	− 4.80 × 10^−3^	9.80 × 10^−3^	688916
rs524533	44240559	C/T	0.42	− 0.10	*9.77 × 10*^*−9*^	− 0.23	1.61 × 10^−3^	− 0.07	0.02	− 0.01	7.80 × 10^−3^	687217

Physical position is based on the human genome GRCH37 assembly. The first and second alleles are the effect and the other alleles. EAF: effect allele frequency in the European population. Beta: regression coefficient of the effect allele. *FHS*, Framingham Heart Study; *WHI-AA*, Women’s Health Initiative–African American sub sample; *KCOS*, Kansas City Osteoporosis Study; *UKB*. UK Biobank. The joint analysis of the GIANT and the UKB samples represents the largest study of BMI, and is used for cross-validation of the association with leg lean mass in the present study. *p* values below the genome-wide significance level (5.0 × 10^−8^) are set in italics. “-,” not available; “p*,” one-sided *p* value

**Fig. 2:**
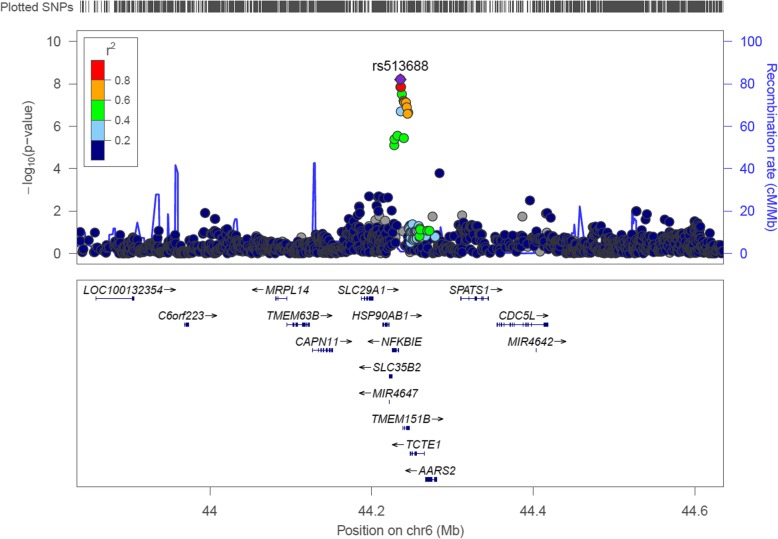
Regional plot of the associated region 6p21.1 in the discovery GWAS Regional plot of the lead SNP rs513688 and its flanking 400 kb region to either side. *X*-axis is chromosome coordinate, and *Y*-axis is minus ten-based logarithmic *p* value

Of the 4 SNPs at 6p21.1, the lead one is rs551145, which is a common (MAF = 0.25) and imputed SNP with high imputation certainty (imputation *r*^2^ = 0.88). Mean (s.d.) raw leg lean mass in the CC, CT, and TT genotype groups are 15.49 (0.08), 15.09 (0.08), and 14.56 (0.10) kilograms, respectively, corresponding to an approximate 0.47 kilogram decrease per allele T. A regional plot of this region is displayed in Fig. 2.

### Linkage disequilibrium analysis

**Fig. 3:**
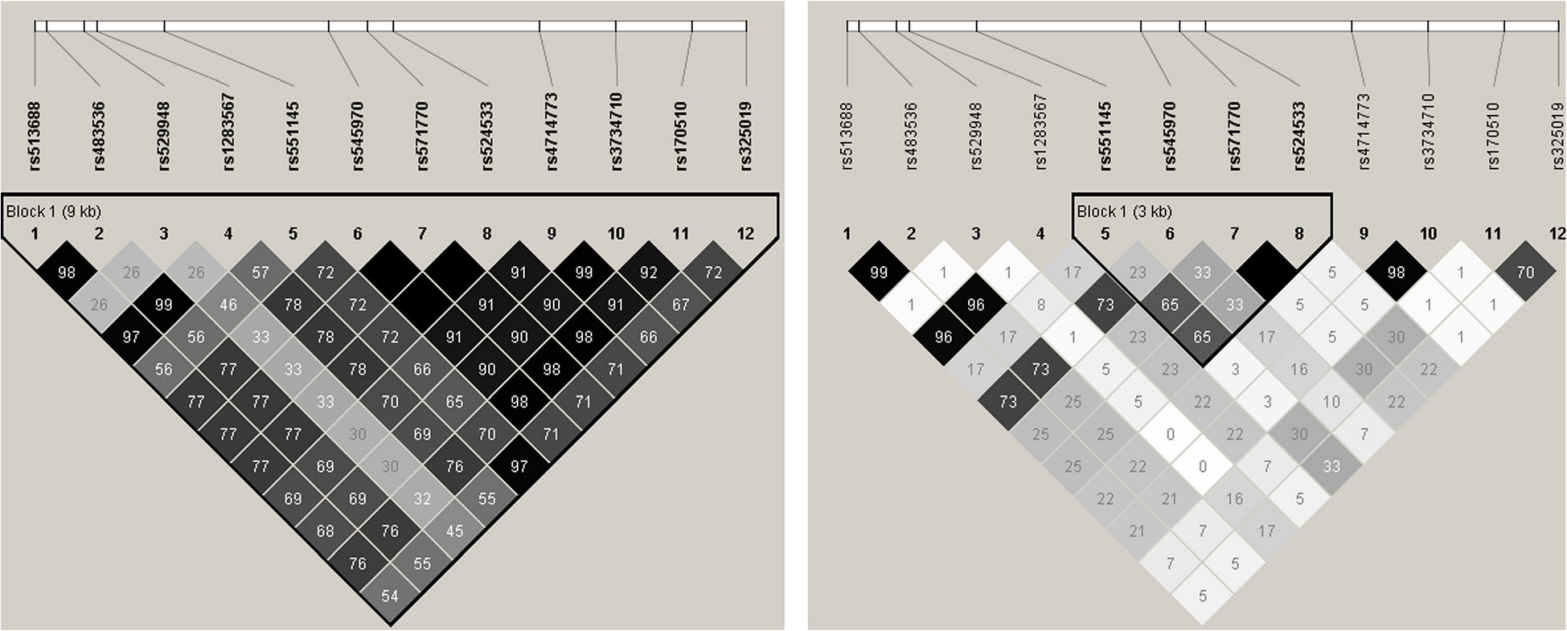
LD pattern of the 12 associated SNP at 6p21.1 Phased haplotypes were retrieved from the 503 individuals of European population (left) or the 319 individuals of African population (right) of the 1000 Genomes Project. LD plot was displayed with Haploview [24]

We explored the linkage disequilibrium (LD) relationship between each pair of the 12 SNPs at 6p21.1 in the European and African populations respectively. The LD structures, as plotted by Haploview [24], are displayed in Fig. 3. The 4 replicated SNPs (rs551145, rs524533, rs571770, and rs545970) are in strong LD with each other in both the European and the African populations, but the LD patterns between them and the other SNPs vary between the two populations. In European population, all SNPs are categorized into one single haplotype block with strong LD structure (*r*^2^ = 0.72–1.00, Fig. 3 left). In African population, on the other hand, the LD levels between the 4 SNPs and the others are weaker (Fig. 3 right).

### Cross-validation in BMI summary results

Fat-adjusted leg lean mass is moderately correlated with BMI. In the discovery FHS sample, their correlation coefficient is 0.23. Therefore, BMI is valid to partially cross-validate the association for leg lean mass. To strengthen the evidence of association, we looked up the association signals of the 4 SNPs in the BMI summary results released by the GIANT consortium, the largest GWAS summary results for BMI to date by integrating the GIANT and the UK Biobank (UKB) participants (*N* ~ 700,000) [25]. rs551145 is not present in the BMI results. All the other 3 SNPs are nominally significant (rs524533 *p* = 7.8 × 10^−3^, rs571770 *p* = 9.8 × 10^−3^, and rs545970 *p* = 7.0 × 10^−3^). The effect direction is also consistent in the sense that the phenotype-increasing alleles are same in both studies. At rs524533, for example, allele T corresponds to both increased BMI and increased leg lean mass.

### Cis-eQTL analysis

We performed cis-eQTL analysis to highlight associated genes. Three of the 4 associated SNPs are located in the intron of transmembrane protein 151B (*TMEM151B*), while rs551145 is located in its promoter region. In the study of peripheral blood by Westra et al. [23], polymorphisms at 3 of the 4 SNPs are associated with the expression of a nearby gene nuclear factor kappa-B inhibitor epsilon (*NFKBIE*, rs524533 *p* = 1.82 × 10^−3^, rs571770 *p* = 2.27 × 10^−3^, rs545970 *p* = 2.43 × 10^−3^), but not with *TMEM151B* (4.7 kb apart from *NFKBIE*). rs551145 is not present in the cis-eQTL summary results. In the GTEx datasets, the associations with *NFKBIE* in the skeletal muscle tissue are observed too, though the signals are a little weaker (rs524533 *p* = 0.09, rs571770 *p* = 0.08, rs545970 *p* = 0.04, rs551145 *p* = 0.05).

### EMSA

**Fig. 4:**
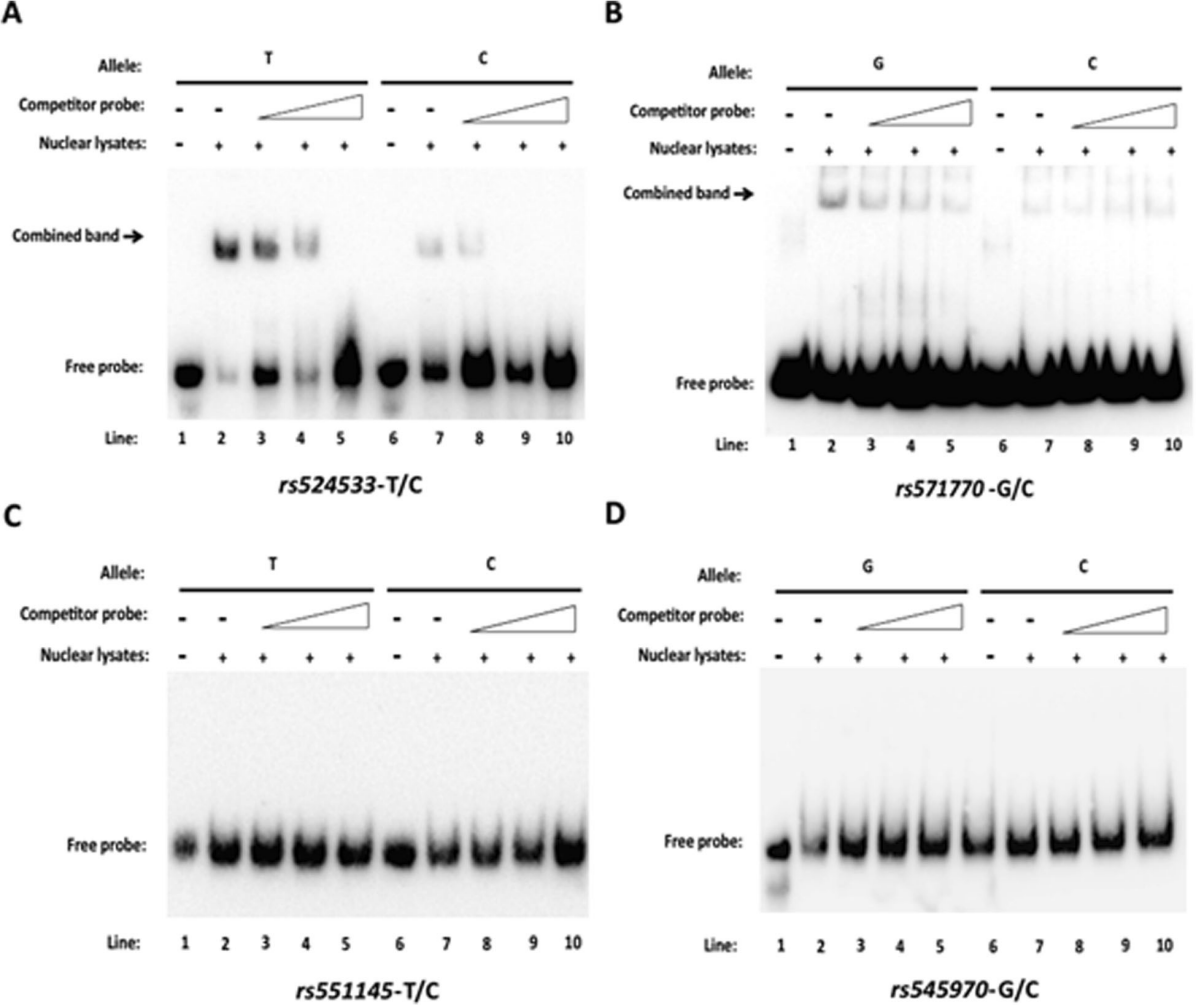
EMSA assay **a–d** EMSA results for rs524533, rs571770, rs551145, and rs545970, respectively. For both alleles at each SNP, five conditions were conducted: (1) negative control in which neither competitor probe nor nucleoproteins were added (lanes 1 and 6); (2) binding reaction in which labeled probe and nucleoproteins were added (lanes 2 and 7); (3) competition reaction in which 2-fold excess of competitor probe were added (lanes 3 and 8); (4) competition reaction in which 10-fold excess of competitor probe were added (lanes 4 and 9); and (5) competition reaction in which 50-fold excess of competitor probe were added (lanes 5 and 10). Only rs524533 and rs571770 showed allelic specific binding affinity to some unknown nuclear protein, while rs551145 and rs545970 did not

We performed EMSA to assess whether the 4 identified SNPs had the ability to combine with certain transcription factor in an allelic specific manner. A 93 bp oligonucleotide containing major or minor allele of each of the 4 SNPs was labeled with biotin and co-incubated with nucleoproteins extracted from C2C12 myoblast cells, and then subjected to EMSA. As shown in Fig. 4, of the 4 SNP, rs524533 and rs571770 show shifted bands at the same migration position for the two alleles, while the other two SNPs rs551145 and rs545970 show no band. This implies that the former two SNPs lie in functional region that combine with transcription factor while the latter two SNPs are merely non-functional tag SNPs. Moreover, the combining affinity differs between the reference and alternative alleles for both SNPs. For example, for rs524533, the band grey level gets obviously weaker when the allele changes from T (lane 2) to C (lane 7). The level also gets weaker with increased ratio of unlabeled to labeled probe concentration for both alleles, and ultimately eliminates completely at a ratio 50:1 (lanes 5 versus 10), implying that the binding is specific to the sequence motif containing the target SNP.

### Dual-luciferase reporter assay

**Fig. 5:**
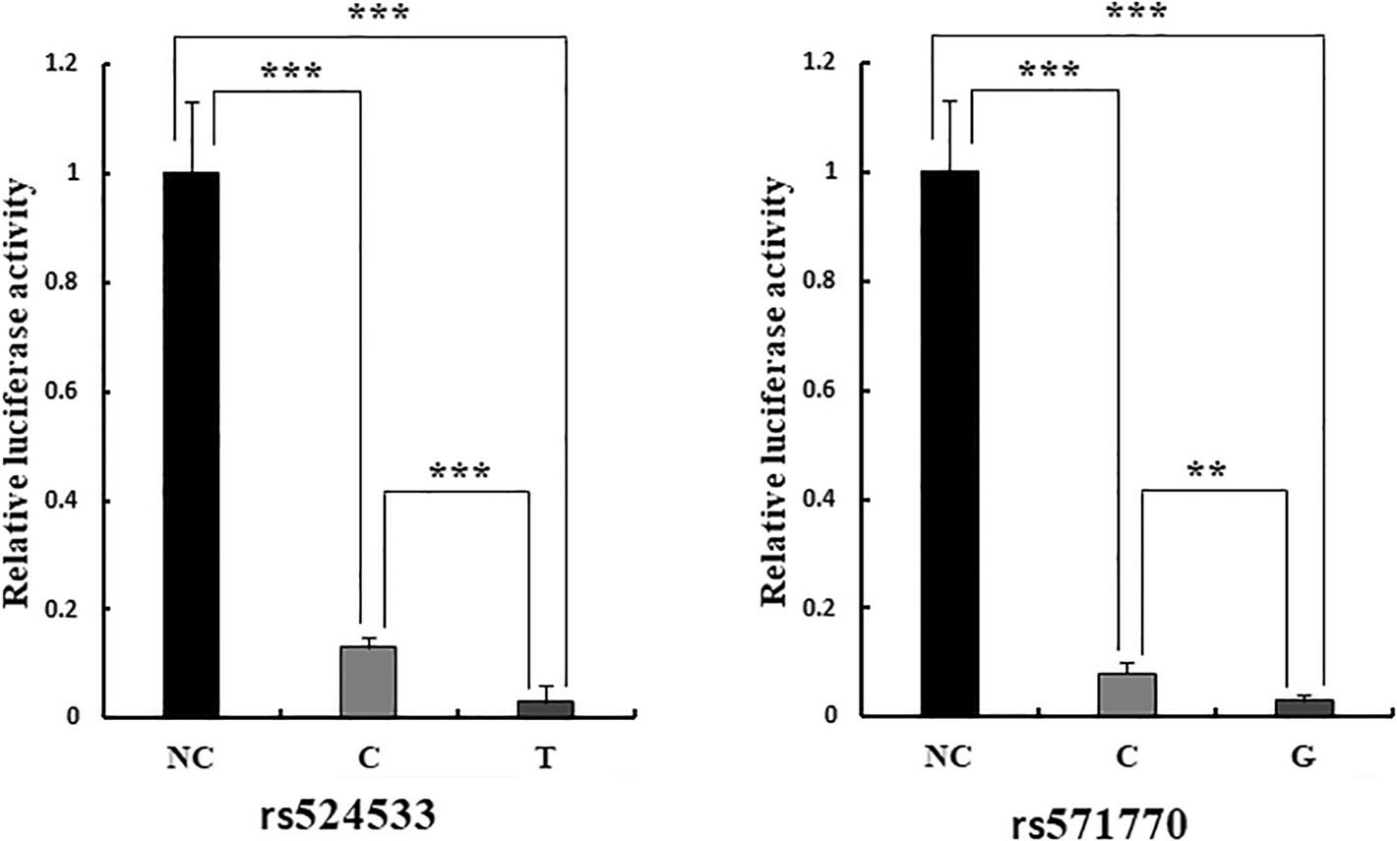
Dual-luciferase reporter assay. The pGL3-promoter vector was set as negative control (NC). Four replicates were conducted under each condition. Compared with the NC vector, the vector containing either rs524533 or rs571770 largely decreases luciferase expression (*p* = 1.07 × 10^−5^–5.33 × 10^−6^). At rs524533, the decrease for allele C construct is less than that for allele T construct by 4.7-fold (*p* = 8.41 × 10^−4^). At rs571770, the decrease for allele C construct is less than that for allele G construct by 2.5-fold (*p* = 4.09 × 10^−3^). **p < 0.01; ***p < 0.001

Based on the EMSA results, we further explored the regulating pattern of two SNPs rs524533 and rs571770 to gene expression in C2C12 cells by the dual-luciferase reporter assay. The results are displayed in Fig. 5. It is clear that compared with the pGL3-promoter plasmid, the plasmid containing either SNP reduces luciferase expression by up to over 30-fold (*p* = 1.07 × 10^−5^–5.33 × 10^−6^), implying that both SNPs repress gene expression. Furthermore, allele T at rs524533 and allele G at rs571770 have less luciferase expression than allele C and allele C (*p* = 8.41 × 10^−4^ and 4.09 × 10^−3^), respectively. This demonstrates that the regulation patterns are allelic specific, consistent with the GWAS and the EMSA results.

## Discussion

In this study, we have performed a genome-wide association study of fat-adjusted leg lean mass in 6587 participants from the FHS, and have carried out replications in two independent samples and in the largest summary results of BMI. A series of investigations, including cis-eQTL analysis, EMSA, and dual-luciferase reporter assay, have been conducted to explore the function of the identified variants. Our results showed two correlated functional SNPs rs524533 and rs571770 at 6p21.1 were associated with leg lean mass after adjustment by leg fat mass.

A total of 12 SNPs at 6p21.1 were significant at the GWS level in the discovery FHS sample. Of them, up to 10 were nominally significant (*p* < 0.05) in the KCOS sample of the same European population. Nonetheless, only 4 were nominally significant in the WHI-AA sample of the different African-American population. The reduced number of replicated variants in a different population is likely due to the different LD patterns between different populations, as shown by our LD analysis. Using trans-ethnic populations of different LD patterns provides valuable information for fine-mapping presumably causal variants [26]. In this part, the 4 identified variants, rs551145, rs524533, rs571770, and rs545970, are more likely to be causal than their neighboring SNPs that were not replicated in the African-American population.

rs551145 is located in the intergenic region of *TMEM151B* and *NFKBIE* gene. The remaining three SNPs (rs545970, rs571770, and rs524533) are all located in the intron region of *TEME151B*, but are close to *NFKBIE* (6.1–7.0 kb apart). Cis-eQTL analysis from two large-scale datasets has provided evidence that polymorphisms of both identified SNPs rs524533 and rs571770 are associated with the expression of *NFKBIE*, instead of the closest gene *TMEM151B*.

*NFKBIE* encodes a protein that binds to components of nuclear factor kappa-B (*NFKB*), trapping the complex in the cytoplasm and preventing it from activating genes in the nucleus. Atrophy and consequent muscle loss in skeletal muscle can occur through activation of the *NFKB* signaling pathway including *NFKBIE* [27]. In rat, *NFKBIE* mRNA expression correlates with different levels of muscle wasting [28]. Variants around *NFKBIE* are reported to be associated with rheumatoid arthritis susceptibility [29].

The EMSA offers a crude visualization of DNA-protein interaction at the protein level. Despite not being able to identify the specific binding protein, our results point to a logical path for the future exploration of our investigation. In Fig. 4a, the free probe in lanes 4 and 9 was lighter than lanes 2 and 3, and 7 and 8, respectively. This is probably due to an experimental flaw, as the volume of reaction reagent was equal in all lanes. We validated the results of EMSA by dual-luciferase assay, which measures transcription activity of DNA-protein complex. We inserted the DNA sequence of the two SNPs rs524533 and rs571770 into the pGL3-promoter plasmid and found that both SNPs downregulated luciferase expression by repressing promoter activity. Our observation of allelic specific regulation was consistent with the GWAS results, the cis-eQTL analysis, and the EMSA results.

Interestingly, we found that though rs524533 and rs571770 are in nearly complete LD with each other, they played an independent regulatory role. This is contrary to the commonly held view, which assumes that there is only one causal variant among a cluster of correlated variants while the signals from the other variants are due to the strong LD patterns with the causal variants. Notably, such biologically independent association signals could not be distinguished by statistical analysis. If true, then the existence of multiple biologically independent variants may offer a new source of missing heritability for complex traits.

Certain limitations exist in the present study. First, we did not explore the joint effect of rs524533 and rs571770 by a haplotype analysis. This is partly because both SNPs were imputed so that the haplotype is difficult to accurately construct. Second, the EMSA assay could only qualitatively visualize the binding affinity to unknown transcription factors, but could not identify the specific transcription factor. Experiments with finer resolution, such as super-sift assay with a set of hypothesized protein antibodies, may identify the specific transcription factor, which is out of the scope of the present study.

## Conclusion

In conclusion, through a GWAS followed by a series of bioinformatic and biological studies, we have convincingly identified two correlated SNPs rs524533 and rs571770 at 6p21.1 locus associated with leg lean mass. Both SNPs show a functional role for lean mass development. Our findings provide useful insights that enhance our understanding of molecular basis of lean mass development and the genetic pathogenesis of sarcopenia, but also provide valuable candidate genes for further functional studies.

## Data Availability

Summary results are available upon request to the corresponding author.

## Abbreviations

BMI: Body mass index
dbGAP: Database of genotype and phenotype
DIV: Deletion/insertion variant
dsDNA: Double-strand DNA
DXA: Dual-energy X-ray absorptiometry
EMSA: Electrophoretic mobility shift assay
FBM: Fat body mass
FHS: Framingham Heart Study
GWAS: Genome-wide association study
HWE: Hardy–Weinberg equilibrium
KCOS: Kansas City Osteoporosis Study
LBM: Lean body mass
LD: Linkage disequilibrium
NFKB: Nuclear factor kappa-B
NFKBIE: Nuclear factor kappa-B inhibitor epsilon
QC: Quality control
TBE: Tris-borate-EDTA
TMEM151B: Transmembrane protein 151B
TRHR: Thyrotropin-releasing hormone receptor
WHI-AA: Women’s Health Initiative–African American
